# American Academy of Dental Science

**Published:** 1892-10

**Authors:** 

**Affiliations:** American Academy of Dental Sciences


### AMERICAN ACADEMY OF DENTAL SCIENCE.

The regular monthly meeting of the American Academy of Dental Science was held in the Boston Medical Library Association rooms on May 4, 1892, President Brackett in the chair.

The paper of the evening was read by Charles E. Francis, D.D.S., M.D.S., of New York; subject, "The Hygiene of the Mouth."

(For Dr. Francis's paper, see page 724.)

### DISCUSSION.

*Dr. Fillebrown.*—I heartily commend Dr. Francis's paper. It is upon a subject which, to be sure, is familiar, but one which gets much neglected at times. His instructions to his patients are very valuable.

I was glad to hear his mention of the use of iodine as a cleanser of the teeth. This use of iodine is not so general as it should be. I once advocated its use in an article and was criticised by *The Journal of the British Dental Association.* This past year one of my patients, whose teeth I have had the care of for many years, was in Europe, and called on one of our prominent American dentists there and had her teeth cleansed. During the process she asked him if he did not use iodine. "Why, no," said he; "it would ruin the teeth." The lady laughed and told him that she had had it used frequently and had yet to see any harm from it. I have proved that it is not injurious. I put a tooth in a bottle containing the tincture of iodine and allowed it to remain there for three or four months. I then removed the tooth and let it soak in water to remove the iodine, and then sawed the tooth completely in two. After soaking for nearly four months in the tincture of iodine, the tooth was just as sound and perfect as it ever was. If there is any member present who does not use iodine in the way described by Dr. Francis, I hope that he will try it at once, and advise his friends to do the same.

*Dr. Andrews.*—I wish to say that I have found it excellent in removing green stains. I am in the habit of using, also, peroxide of hydrogen together with the pumice and the iodine, and I think with those three materials you can meet any case.

*Dr. Banfield.*—Can any one tell me what compound forms in connection with this green stain?

*Dr. Williams.*—It has been known for some time that iodine, followed by ammonia, would take off nitrate of silver stains. I have forgotten just the chemical combination, but there is an accurate scientific reason for it.

I was much pleased with the paper; there were one or two points in it that I was particularly glad to hear mentioned. One of them was the fact of the dentist being blamed for more than he is responsible for. The essayist spoke of the importance of faithfulness in cleaning, etc., but how long do the cleansed teeth remain clean? I could not help recalling a case of old Dr. Joshua Tucker which he related some thirty years ago. He had put in some fillings and cleaned the teeth of a gentleman, leaving the mouth in very good condition. In the course of six months or a year the patient returned, and in looking his teeth over he found that one had decayed in an approximal cavity where he had placed a filling, and the patient began at once to blame him for not filling the tooth properly. The old doctor said to him, "The mischief started there and I stopped the decay; now it has started again in the same place and from exactly the same cause. Do you think I can do better than the Almighty?"

I always think of that when I hear dentists bemoaning so-called failures as if from lack of faithfulness in filling teeth, when, in many cases, it was from no fault of the operator, but came from just the same cause as produced the original decay. We should not always be blaming ourselves or others when we see mischief rampant.

In the matter of brushing, sometimes patients in their zeal to use the brush will get a tension on the lips and muscles of the mouth and face which prevents the free motion of the brush over all the teeth. I always tell them to let their muscles hang as lax as if they were going to sleep, and then they can get their brush anywhere, and will be likely to brush more thoroughly. I prefer a brush with fine but elastic bristles, not too closely set. This has the effect of a moderately soft brush, and not the harshness of a stiff brush. There is one great fundamental fact to be borne in mind in allowing children, or even adults, to use too stiff a brush, —they insensibly, if not consciously, avoid getting it near the gums. By using a softer one there is no danger of injuring the gums, and it can, with the proper motion, be used all over the teeth. The motion that I direct is a circular one, corresponding nearly to the diameter of the teeth. In this way you get a continuous friction into the spaces and around the curves of the gum. I also recommend to patients the use of the rapier-pointed quill toothpick, not too stout, but so thin that it will soon soften in using. Of course there are spaces that you cannot get at with this, and you have to use floss silk. But I recommend this kind of quill because it is so well adapted to reach up under the gum. With the obtuse wooden toothpick you press material up into those crevices and keep it there.

*Dr. Francis.*—I have used borax successfully, especially in connection with inflamed gums, and in many cases of pyorrhœa alveolaris. It is an antacid, and, to a certain extent, an antiseptic. I direct patients to wet the tip of the brush, and pick up some of the borax with it and place it on the gums; wait a few seconds until the crystals are partially dissolved, and then whirl the brush without scratching the gums. The borax dissolves and penetrates the viscid material that lies directly at the necks of the teeth, and you can then brush it away.

*Dr. Andrews.*—There is one point that I did not quite catch. The essayist spoke of the iodide of zinc for congested gums. I should like to know a little more about this material and its action.

*Dr. Francis.*—Iodide of zinc is one of the best astringents that can be used in the mouth. It was first suggested to me by Dr. R. P. Lincoln, a New York specialist of throat and lung diseases. He told me that he applied it in throat troubles, and it occurred to me that it might prove effective as an application to inflamed gums.

You can easily prepare the iodide of zinc. It is a combination of compound tincture of iodine and sulphate of zinc. The formula of the former may be found in the "United States Dispensatory." The sulphate of zinc should first be a saturated solution. They must be carefully combined, otherwise the iodine and zinc will both precipitate. Dilute the compound tincture one-quarter with alcohol, and dilute the solution of zinc three-quarters with water. Pour slowly, drop by drop, the solution of zinc into any given quantity of the solution of iodine until the iodine shows signs of precipitation. I use it much in cases of socket-disease, and find it an excellent preparation to inject into the pulp-canals of roots in treating alveolar abscess.

*Dr. Williams.*—I have had some experience with that material myself, using it with great benefit in some cases of pyorrhœa alveolaris.

Dr. Fillebrown's mention of the dentist who thought iodine injurious reminded me of a similar experience that I had in relation to the use of borax. I had used it for some years, and a patient of mine, who understood its use, recommended it to a friend of hers. Afterwards that friend had occasion to go to her dentist and she mentioned, casually, that she had been using borax. "Why," said he, "you will ruin your teeth with borax." In November, 1889, I put some cuspid teeth in a bottle containing a saturated solution of borax. I looked at them yesterday, and the teeth were unchanged.

*Dr. Clapp.*—One of the best results of our meetings is in keeping us up to concert-pitch for our daily work. We cleanse teeth day after day, and I, for one, sometimes get tired of it. Certainly, if there is anything that needs doing thoroughly, it is the cleansing of teeth, and it is well for us often to consider this matter.

I use most of the methods Dr. Francis has spoken of, and in addition floss silk, and when I am not too tired I instruct patients how to use the silk themselves. Almost invariably the question asked by the patient while you are cleansing the teeth is, "How can I keep this tartar off my teeth?" I reply that I know of but one way, and that is by using floss silk. Then, giving the patient a mirror, I demonstrate its use. Another time for using floss silk is after using pumice. It removes all the particles of pumice and calculus that remain between the teeth, and leaves the mouth in a much more comfortable condition. The syringe will not remove all of the débris.

Another preparation that I have been in the habit of using in the place of iodine is a formula composed of tincture of iodine and glycerin, equal parts; to one ounce of this mixture add ten drops of carbolic acid (deliquesced crystals). I don't know that this will act quite as readily on the green stains as the plain tincture of iodine, but it is much less disagreeable to the patient, and it will act nearly as well. It is also remarkably adapted for congested and inflamed gums, and it is almost a specific for cracked lips. We have a large number of patients coming to us in the winter-time with cracked lips, and it is certainly a very painful and disagreeable trouble.

Dr. Francis has spoken of listerine. I would like to ask him if he fears anything from its acid nature. It has quite a strong acid reaction from the boracic acid contained in it. I am in the habit of using it, and in the mouths of patients who are troubled with what we call tartar I have recommended it freely.

*Dr. Francis.*—I believe that a single drop of milk, if left to ferment in the mouth over night, will work more mischief to the teeth than could possibly result from a free use of listerine. I never like to advertise patent nostrums, but I have witnessed such excellent results from the use of this preparation that I feel impelled to refer to it favorably. I know of no other mouth-wash which has proved so reliable, and in some cases where I have found teeth in such a condition that I almost despaired of saving them, they have been kept well by the use of listerine. Some of the best-preserved teeth that I have seen are in the mouths of those who habitually use it.

It has been remarked that the application of iodine to the teeth is unpleasant to some persons; but not once in fifty times where I have used it has any such complaint been made. I have, however, in a number of instances, had patients speak of it as a "pleasant-tasting" application. It is invaluable when cleansing teeth for children, because it shows up the stains and renders them easy to remove.

Viewing the case either theoretically or practically, we need fear no harm from the use of either iodine or listerine in the mouth. If any person present has never tried iodine for cleansing teeth, I would advise him to do so. It will disclose stains not easily discovered without applying it.

*President Brackett.*—I remember a score of years ago, in my student days, it was my privilege and advantage to have some excellent instruction and advice upon the subject of cleansing teeth. I am sure it will gratify every one of us to hear Dr. Moffatt speak on this subject.

*Dr. Moffatt.*—The subject of the cleansing of patients' teeth by the dentist has always been one of great importance in any practice. I have always insisted that the dentist should put every one of his patients through a course of careful inspection for the cleansing of their teeth, some needing it once a year, some twice a year, others three or four times a year. When I held the chair of Operative Dentistry in the Harvard Dental School, I endeavored to instruct the students as well as I could upon the importance of this subject.

I had a system of cleansing the teeth and a set of suitable instruments for doing so. Commencing with the lower central incisors, I passed around on one side as far as the wisdom-tooth, then in the same way with the other side, and then proceeded to the upper ones, carefully inspecting every tooth, and getting it as nearly clean as possible.

Dr. Francis made one suggestion in his paper that I do not quite agree with, and that is with regard to the free use of tepid water during the operation of cleansing the teeth. There is more or less bleeding at the time, but I think the use of so much water is a little injurious: it dilutes the fluids of the mouth too much, and also the blood. I generally get along as much as possible without it, allowing the patient to free the mouth with saliva. At the close of my cleansing, then I give a little tepid water and use a little tannin.

I quite agree with him in the use of iodine and of listerine. One thing that I have generally used in connection with listerine is a little glycerin.

Some one asked what was the combination produced by the use of iodine in removing stains. I think I can make that clear. These stains are usually produced by iron. By the application of iodine, we form the iodide of iron, which is soluble and easily removed by polishing.

The cleansing of patients' teeth is one of the most important duties of the dentist, and most dentists, I am afraid, shirk it a little. I always give patients a lecture on the use of the brush and floss silk, and to a business man I always recommend the use of little rubber rings, such as he is apt to use in his business; these will do the work nearly as well as floss silk.

*President Brackett.*—I have been noticing our worthy member from Salem taking notes. Perhaps he will give us the benefit of his ideas.

*Dr. Meriam.*—I like to make notes as I hear things.

Regarding the use of iodine, of course we know that it belongs with bromine and chlorine; all of the group have bleaching properties. I don't know who introduced it in dentistry; I found it in use at the Harvard Dental School in 1871.

*Dr. Moffatt.*—If Dr. Meriam will allow me, I think the credit of introducing it is due to Dr. Thos. B. Hitchcock.

*Dr. Meriam.*—Thank you. I think that should go on record.

There is one thing that should be noticed, and that is the care of children's teeth when they are too small to come to the dentist. The nurse who has the care of the children can take one tooth at a time and attend to it, whereas in our office it would be too much for the little one's patience, and we cannot afford the time. For that purpose the best thing to use is a piece of rattan, cut to a point like a pen. I think the use of rattan for cleansing teeth came from Dr. Williams, and I have now several hundred rods of it in my cellar that I use for gardening and other purposes. I think it can be had at almost any furniture-store.

Another thing that I like to use is elder piths. They can be bought in bunches and are soft as chamois skin, and little pieces can be used in the final polishing.

Following the pumice, we can use oxide of tin. That was formerly used a great deal, and I always keep some on hand. I get it from Metcalf's in the wholesale department, and if it is not in any ordinary chemist's list, you can get it by sending there.

The rapidity with which anything acts in the mouth should be remembered. Heat and moisture are the great factors in the germination of plant life, and as every growth in the mouth so far discovered has been vegetable, we should keep that in mind. If you will notice a glass of water standing in a room, you will see how quickly the sides of it become coated. You will also notice how quickly the spores of ferns will germinate in a hot-house, small ferns often coating the sides of pots. I have often thought that as spores of plant life develop so rapidly, how important is it that we should use mild alkalies in cutting or dissolving away the mucous coating which is so easily formed.

There is one thing to be remembered, however, and that is the increased power of dilute medicines in proportion to that of their powder form. If we are to use anything as a medicine, I prefer a solution; but if we want it as a polisher, then I would use powder. Two years ago I completely cured a case of erosion by the use of a wash. All the front teeth were being rapidly eroded. I recommended the use of alcohol, the instructions being to get a burning in the mouth twice a day. The object of this was to reach the mucous glands that were giving out acid, and to give them a stinging so as to change the character of the fluids.

*Dr. Clapp.*—I would like to speak of a different use of iodine, and ask if any one has ever employed it in this way. During the year 1869 I had the pleasure of being, for about six months, in the office of the late Dr. Coffin, in London, and in the excavation of cavities he always used iodine. When the cavity was partially excavated, he introduced in it a little of the tincture of iodine, thereby getting the same result which we get on the stains of the teeth when we use it for cleansing. He seemed to think that if there was anything remaining in the cavity that was devitalized it would be colored and detected at once. I don't think he ever excavated a cavity without employing it.

*Dr. Williams.*—Did he depend upon excavation to remove the stain of the iodine, or did he use something for that purpose?

*Dr. Clapp.*—Nothing more than water. As I understand it, iodine will not discolor sound dentine; the color remains only in the dead portion, which of course has to be excavated.

*Dr. Meriam.*—One thing I omitted to mention, and that was pine-sticks. An excellent way to get them is to use the unpainted pot-plant labels. They are made of soft pine, usually straight and clean, and I get perhaps five hundred of them for a small sum, from seedsmen.

*Dr. Eames.*—As a finisher to my operation of cleansing I have used, with great satisfaction, fine, antiseptic, warm spray, given with a good deal of force such as you can get with a compressed air-tank or the foot-bellows. This is like a searching wind and rain which finds access to otherwise inaccessible parts. This warm spray used with force seems to drive all particles from the neighborhood of the teeth. I have experimented with the hand-ball atomizer to get a spray which patients might use, but it does not give sufficient force to accomplish the object.

*Dr. Smith.*—The essayist spoke this evening of passing the brush over Castile soap before using tooth-powder. I have felt that the use of Castile soap was injurious in many mouths. I came to this conclusion from observation, and also find that Dr. Garretson speaks of soap as having a tendency to produce recession of the gums. I do not know of a case that has come to my notice of extreme sensitiveness about the necks of the teeth where the patient would not answer "Yes" to the question, "Do you use soap?" I have prohibited in those cases the use of soap, and in many the sensitiveness has entirely disappeared without any treatment whatever. Dr. Shaw, whom some of you know has experimented in that direction on his own teeth, says that if he uses a tooth-powder with the slightest trace of soap in it his teeth will soon become so sensitive that the drawing in of air will produce pain, and that by changing for a powder in which there is no soap present, and with no other treatment whatever, that sensitiveness entirely disappears. I believe that dentists recommend Castile soap altogether too freely.

*Dr. Moffatt.*—I have had patients ask me, "What makes my teeth look so yellow and dingy?" I reply, "If you will give up using soap and get some powder that has a little friction, it will probably improve the appearance of your teeth." The fact is, that soap makes the mucous secretions of the mouth thick and heavy, and they stick to the teeth, and that covering is not readily removed by the brush, and so the teeth look dirty. The idea of using soap is apparently a very good one, but in the mouth it doesn't work well.

*Dr. Andrews.*—I have found the same trouble with soap that Dr. Smith speaks of: it is likely to produce recession of the gums.

*Dr. Williams.*—You can over-stimulate the gums and make them recede by too much brushing. In regard to the discoloration of the teeth referred to, you cannot keep them white unless you use some powder with the soap. The soap acts as a lubricator for the brush.

*Dr. Harris.*—I would like to hear a few words on this subject from Dr. Stanton, President of the Harvard Odontological Society.

*Dr. Stanton.*—The subject has been so fully discussed that I cannot add to what has already been said. Dr. Andrews spoke of the use of peroxide of hydrogen as one of materials which he uses. I would like to ask if he has noticed any effect from the hydrochloric acid in the peroxide of hydrogen. As I understand it, in the manufacture of peroxide of hydrogen the makers are obliged to acidulate the water with a small quantity of hydrochloric acid. My attention was called to the matter by one of my patients, who asked if I thought it injurious for her daughter to gargle her throat with peroxide of hydrogen. The child's teeth were not in very good condition around the margins of the gum, and I suggested that she bring me a small quantity of what she was using. She did so, and I tested it. It surely contained a small quantity of acid, although it was a difficult thing for me to get an acid reaction. As an experiment, I then tested some of Marchand's, such as we get from the stores, and I found that it was very strongly acid, and it occurred to me that in children's teeth, where they were disposed to decay around the margins of the gum, it might possibly be injurious.

*Dr. Andrews.*—I was cautioned in the use of peroxide of hydrogen by what Dr. Rollins said in regard to it. I have used it with care, and, as far as I can see, there has been no injurious action as a result.

As to listerine, one can see how very acid it is when they test it. It turns blue litmus-paper red, and I have been cautious in recommending it, telling patients to use it no stronger than one-third listerine and two-thirds water. Recently I have been using phenol sodique a good deal instead of listerine, and I like it quite as well, or better. It is an antiseptic, and I use it for retarding the action and growth of germs in the mouth and teeth.

*Dr. Meriam.*—The use of these things is very general, and the manufacture of them is closely followed up by the various druggists, so that it is quite easy to obtain the various formulas for listerine, etc. It seems to me that the profession is getting numerous enough now to have a book of formulas, as some of the hospitals have, and it would seem as though we ought to have preparations made for us as we wish them, and see to it that they are not injurious. For instance, we could have a listerine possessing its present virtues with the acid left out.

William H. Potter, D.M.D.,
*Editor American Academy of Dental Sciences.*

